# The plants, rituals and spells that 'cured' helminthiasis in Sicily

**DOI:** 10.1186/1746-4269-4-21

**Published:** 2008-09-29

**Authors:** Mariangela Napoli

**Affiliations:** 1 Dipartimento di Botanica, Università di Catania, Via A. Longo 19 – 95125 – Catania, Italy

## Abstract

**Background:**

The author reports on the plants, rituals and spells used against worms and the so-called *scantu *(fright) in some areas of Sicily. The work is based on ethnobotanical research carried out, prevalently, between 2002-2006, in some areas of Eastern, South-Eastern, North-Central and South-Central Sicily.

**Methods:**

This research is based on dialogue. Senior 'healers' were contacted; furthermore, doctors, teachers, farmers and in general 'experts' with herbs and 'magic' rituals. Information was collected about the way the plants of folk medicine are prepared. The interviewees were also invited to recite prayers and spells against helminthiasis.

**Results:**

The author has highlighted the importance of how, in some parts of Sicily, some ailments like helminthiasis and other correlated pathologies like *scantu *are 'treated' and, especially within the rural social classes, by folk medicine remedies, herbal practises, particular prayers, rituals and spells.

**Conclusion:**

As regards health/illness, it should be noted that in the last ten years conventional medicine has provided very satisfactory results even resolving potentially mortal pathologies. However, in certain social classes, there is no real collaboration between conventional and folk medicine; so for some senior citizens, the 'healer' with his rituals and empirical and magical herbs is still the person to turn to for the 'cure' of particular ailments. Interest in these practises from ancestral heritage in an advanced country like Italy, is only relevant if the aim is to recoup a cultural identity which is already in decline.

It is significant to report a piece: on 14 October 2007 the news on a well-known national Italian TV channel reported an interview with a 94 year-old man from Arbatax (Sardinia) referred to as a 'healer' because both his townspeople and others from all over the world go to him for his cures. He is not paid except in kind and has been known to cure St. Anthony's fire, burns, scalding and marine fungal infections, by smearing his saliva over the infected part and reciting 'special words'.

## Background

This work, carried out by the Botany Department of the University of Catania, forms part of a series of ethnobotanical investigations in several zones of Eastern Sicily (Mount Etna [1,2]); North-Eastern (Nebrodi Mountains [3]); South-Eastern Sicily (Caltagirone and Ragusa), North-Central Sicily (Madonie mountains) and South-Central Sicily (Caltanisetta) [4-8].

The research highlights how even nowadays, in Sicily, above all in some inland areas and rural communities, an illness like helminthiasis is still considered 'magical' according to some popular beliefs and is cured by either remedies from medicine and folk herbal preparations, or by certain practises (of 'healers') like prayers or 'medicinal spells'. Among these the most common are those which *pircantari *or *ciarmari i vermi *where 'pircantari' means to recite a spell to keep away the bad spirits which carry disease, and 'ciarmari i vermi' means to carry out magical practises to cure children of worms [9].

It has been identified that [10] illnesses from 'magical' or spiritual causes can be cured by means of either oral formulae together with biological and non-biological material (used as medicines or applied as ritual objects) or oral formulae with Western pharmaceutical products [10].

If the therapies do not work within nine days in the case of spiritual illness the patient can look for another 'healer' to find a different cause of the illness. The failure of the first healer is proof that he did not identify the reason for the onset of the illness. However, if even the second cycle of treatment does not produce the desired result, most people then refer to conventional medicine [10].

As regards helminthiasis specifically, it should be remembered that conventionally, it is a parasitosis due to which some part of the body is infested by worms. In general, helminths are found in the alimentary canal, but could reside in other organs like the liver. In some countries, worms are associated with illnesses which affect various parts of the body; in Austria, for example, four types of worms have been described one of which – considered able to attack the heart – has been held to be particularly dangerous for the general health of the patient [11].

In many areas of Sicily (the Madonìe mountains, Ragusa and Enna), helminthiasis which is commonly contracted in childhood is still today considered a 'psychological' illness. According to certain beliefs, helminthiasis onset in children, follows a strong attack of the jitters (*scantu*) which would cause 'a shaking up of the worms' ('*a-rriminiata rê vièrmi'*) which usually live intertwined in the stomach. This movement would produce symptoms like swelling, stomach pain, vomiting, halitosis, convulsions, persistent cough, sneezing, itchy nose and respiratory problems, due to the disturbed worms climbing along the airways and threatening to suffocate the child ('*fari accupari u picciriddu'*) [4]. However, it seems that children on a diet of sweet food, milk, vegetables and dried fruit are exempt from the malady.

With the jitters or a strong fright as its basis, not surprisingly there are numerous dialectal sayings which refer to strongly shocked people like 'they've made his wicked worms jump' ('*Cci ficiru sautari i vermi maligni'*) or, 'Don't give him the worms!' ('*Nun-cci fari pigghjari na virmina*!') and 'Don't stir up his worms!' ('*Nu-cci fari arriminiari li vermi*'). In adults, worms are less dangerous than for children, but can cause blepharitis, dental decay and otitis [12] whose symptoms are prevalently related to the nervous system which would explain: 'Worms and nerves – they're the same thing!' ('*Vermi e-nnervi sunu tutta na cosa*').

The jitters which follow an emotional outburst is a pathology which as a 'psychological illness' finds its counterpart in the folk medicine of Latin America as "susto" (Portugese and Spanish for 'fright') [13,14], in Sardinia as 'assuto' [15] and as 'assichidu' [16] in the province of Ragusa and other Sicilian provinces.

## Methods

### "Areas analysed"

Research was carried out in the following localities (provinces): Acquedolci, Alcara Li Fusi, Alimena, Caronia, Castel di Lucio, Casalvecchio Siculo, Cesarò, Galati Mamertino, Militello Rosmarino, San Fratello (Messina); Sperlinga, Villarosa, Valguarnera (Enna); Capo Scalambri, Modica, Monterosso Almo, Punta Braccetto, Punta secca (Ragusa); Belpasso, Caltagirone, Sant'Alfio, Santo Pietro di Caltagirone (Catania); Calcarelli, Castellana Sicula, Ganci, Petralia Soprana, Polizzi Generosa (Palermo).

**Figure 1:**
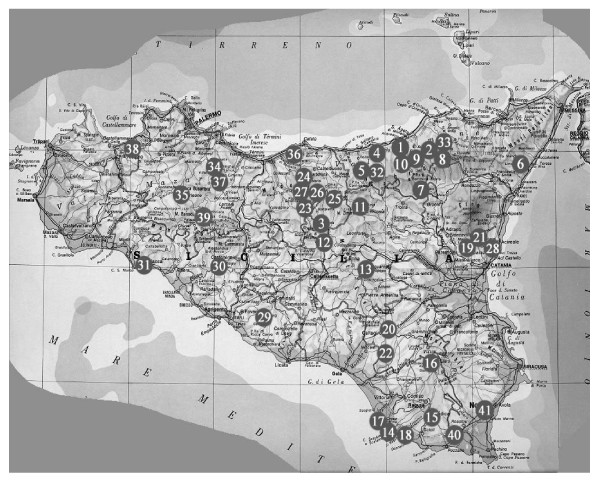
**Map of Sicily reporting analized localities (scale 1:1.000.000):** (1) Acquedolci, (2) Alcara Li Fusi, (3) Alimena, (4) Caronia, (5) Castel di Lucio, (6) Casalvecchio Siculo, (7) Cesarò, (8) Galati Mamertino, (9) Militello Rosmarino, (10) San Fratello, (11) Sperlinga, (12) Villarosa, (13) Valguarnera, (14) Capo Scalambri, (15) Modica, (16) Monterosso Almo, (17) Punta Braccetto, (18) Punta secca, (19) Belpasso, (20) Caltagirone, (21) Sant'Alfio, (22) Santo Pietro di Caltagirone, (23) Calcarelli, (24) Castellana Sicula, (25) Gangi, (26) Petralia Soprana, (27) Polizzi Generosa, (28) Acicatena, (29) Naro, (30) San Biagio Platani, (31) Sciacca, (32) Mistretta, (33) Tortorici, (34) Cefalà Diana, (35) Corleone, (36) Lascari, (37) Mezzojuso, (38) Partinico, (39) Prizzi, (40) Ispica, (41) Noto.

Furthermore, bibliographical references were obtained from (provinces): Acicatena (Catania); Naro, San Biagio Platani, Sciacca (Agrigento); Mistretta, Tortorici (Messina) Cefalà Diana, Corleone, Lascari, Mezzojuso, Monterosso Almo [17], Partinico, Prizzi (Palermo); Ispica (Ragusa); Noto (Siracusa) (Figure 1).

### "Research method"

**Figure 2:**
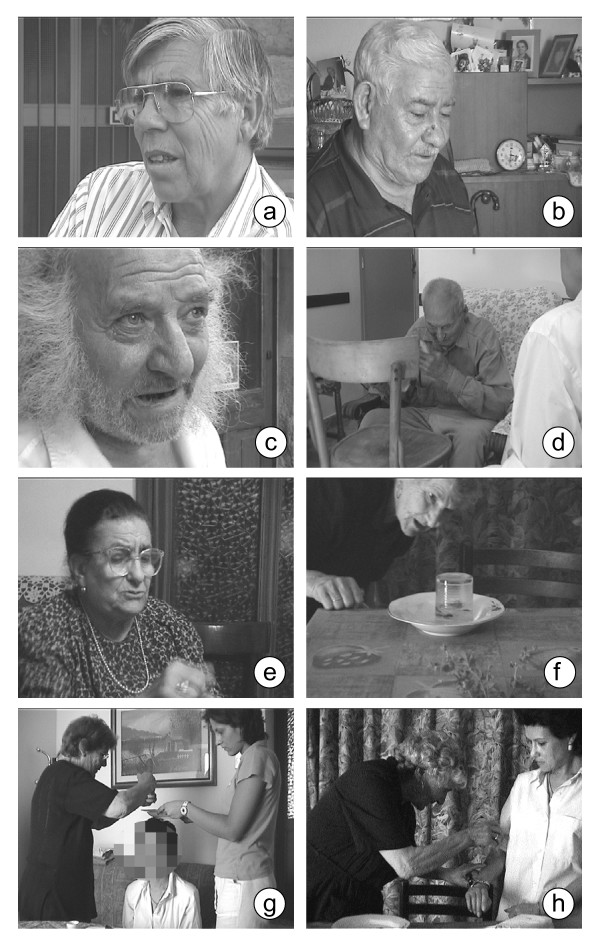
Photos of interviewed 'healers' of some analysed localities.

During several stays in the above mentioned areas, research was carried out in the form of interviews (some videotaped) with local folk who were mostly elderly (men & women) and in particular with those well-known as 'healers' or would-be healers (Figure 2). About twenty-five consenting 'healers' were interviewed. Most, known as [18] 'those who help' – were elderly (60+) unmarried women or married with families. Some were younger (40 – 50): daughters, granddaughters, or anyway relatives or godparents. These women helped out with the rituals. Since they had demonstrated their belief in them, they were asked to comment on their various phases. So, these 'helpers' – speaking in good Italian thanks to a reasonable education – gave a lot of very detailed information about the rituals, the type of patients who seek out elderly 'healers', the frequency of encounters, 'therapy' duration and its outcome.

Some healers were men between seventy and eighty, except for one who was nearly a hundred! They were doctors, pharmacists, teachers, farmers, priests, shopkeepers, and one old colourful second-hand dealer (Madonìe mountains) with an unruly white beard who was an expert with herbs and 'magic' rituals. The figure of healer, still today and not just in the countryside but also in the city, emerged from our interviews as one who offered his services to cure trapped nerves, dislocations and strained muscles ('*sfilatini*'). Moreover, the worm-charmers or *ciarmavermi *(those who practise anti-helminthiasis rituals), other 'specialist' healers of particular illnesses and their helpers were contacted when pathologies other than those for helminthiasis presented like hernias, cuts, abrasions, burns, mycoses and skin eczema.

Various interviewees were asked about the use of plants, their local names and where they grew. They were also asked to recite prayers, songs and spells enacted to cure patients. Some were transcripted directly as they were recited, others were filmed during the ritual. Originally, the transcripts were in Sicilian dialect. In a case of treating helminthiasis in San Fratello with garlic and a spool thread, the prayer (*a-rrazzioni*) recited for the occasion (see below), was transcribed in the local dialect, according to phonetic and handwriting suggestions from a local healer.

Since most patients prefer ritualised treatment at home, we were guests there. In some cases, patients had to travel away from home to the healer's town where we too observed the rituals, sometimes staying for over an hour. The diagnostic process in most cases was based on questioning the patient about their symptoms and careful attention to descriptive detail. If the illness was physically manifested like in dermatitis, a wound, a mycosis or other, the healer accurately examined the infected area annotating the location and symptom type, the period of onset, and its possible regression or reappearance. If helminthiasis was the illness, the healer would ply the intestines to quantify the parasites, localise them and identify their orientation whether curled up (*a-gghiuòmmaru*) or ring-shaped (*a-ccuddùra*). The accompanying administration of phyto-remedies, decoctions, infusions or other folk medicine preparations were also carefully monitored. Whenever possible, the author made sure that the healers' therapies were witnessed in order to personally document and in some cases film the 'cures' prepared for 'healing' helminthiasis.

For the nomenclature of the taxa, 'Flora d'Italia' [19] was the primary reference, a work in three volumes, which provided morphological and bio-ecological information. In some cases, its nomenclature was updated [20].

All the corresponding dialect, common and scientific names are reported in the index. Various Italian texts were consulted for phytotherapeutic, ethnobotanical and dialectal data [9,21-30].

## Results

The results of this research have highlighted how folk beliefs regarding the fascinating study of folk medicine and herbal practises connected with apotropaic therapeutic rituals still play a part today in the cultural heredity of the areas under study and, passed on by word of mouth, still survive in the rural habitat in farming areas. Casting 'spells' (dialectical variations: *ciarmu*, *cirmu*, *cirmata*, *pircantu*, *pircantata*) and reciting particular incantations, words or prayers is considered a medico-magical heredity which is passed on from woman to man and vice versa and can only be practised by the 'disciple' upon the death of the 'master'. To be able to cast a spell, according to a healer in the Madonìe, you would need to visit the trustee of that spell every Friday for seven consecutive Fridays. Healers are not be paid in money at risk of losing their powers but only in kind if offered. Data confirms [18] that healers are paid a small offering made by the family of the patient or with some food, maybe oven-made cakes, or with jams or vegetables.

### "The worms charmer"

To better understand the healer or worms charmer (in dialect) of the previous paragraph it would be opportune to make a brief digression. "In the complex world of occupations involving rituals and therapeutic techniques two figures stand out in (Sicilian) folk culture: the magician and the worms charmer" [31]. The magician possesses and manages the spirits (supernatural beings) making use of them in the practise of his occupation which is to cast or break spells and to cure the evil eye [4,5]. Worms charmers do not control spirits: they are 'those who charm the worms' or 'those who do incantations'. These empirical 'doctors' are also consulted for ailments other than helminthiasis like stomach ache, lumbago, breast blockage, jaundice and sunstroke. Worms charmers can also remove the 'evil eye' and the 'bad mother' which is an infection which, according to popular belief, affects women in labour and manifests itself in hysterical convulsions.

All the ailments treated by worms charmers are considered 'natural' even when spirits are involved. A scare (fright, jitters etc.) is always cited as the precursor to helminthiasis (or other ailment) and could be the consequence of an encounter with the spirits or the entry of a spirit into the body (by inhalation), in the case of a surprise fear (*arrisagghiari*).

In the course of the interviews it became evident that worms charmers receive ample credit from the society in which they operate; generally they are women and all the more believable because they are of that venerable age which carries with it so much more experience. They are so integrated into daily life that they are known by affectionate monikers: Aunty Maria (*a-zza Maridda*), Mrs. Giuseppina (a first name) (*a-ggna Pippina*), and 'mouth of steel'(*Pizzudazzaru*)!

It was also noted that many worms charmer practises could be carried out by cognoscenti but that when the ailment was particularly serious requiring a more personalised therapy the expert healer is always called in. The worms charmer 'cure' consists of a ritual based on incantation and oration, but also a genuine exorcism. The healer bases her diagnosis on symptoms – usually confirming what recounted either with certainty or by supposition on the part of the patient – kneading the ailing part of the body or resorting to remedies like the 'little worm cup'(*cicaredda-ppê viermi*) or the 'little plate for the evil eye'(*u piattinu*). When worms are suspected a small coffee cup whose rim has been coated with garlic and oil is placed rim-down on the patients navel: if the cup (*cicaredda*) sticks the patient has worms.

Having learned how 'to remove the frights' a worms charmer from Lascari went to Christmas midnight mass reciting the prayers he had duly learned. After communion, he paid his penance by having three handfuls of blessed olive leaves and three of palm leaves and a sip of water. After the *Via Crucis *and other orations, the neo-healer went into the countryside, found a thistle with a white worm on it, pulled it out by the roots and rubbed it over both hands. For the next three days he did not eat, drink or sleep but went to communion, continued the penance and said the prayer: "...for friends, for enemies, for the dead, and for the abandoned" [31].

### "The virtue of birth"

Most worms charmers we contacted affirmed to having been chosen in order to pass on 'knowledge' because they were 'gifted' or had a 'virtue of birth' or something acquired through ritual. It was right down to such a virtue that these women affirmed their power to be able to 'perform as specialists'. The 'virtue of birth' can take different forms: one healer said of that herself she 'was born with her umbilical chord around her neck and was baptised on the same bench upon which she was born'. Baptism upon birth is said to confer strength as well as extraordinary powers on whom has had a risky birth.

In some cases, these healers affirmed they could 'beat' psychic ailments like being possessed and pathologies like the 'frights' because they amounted to something magical and so are curable 'magically' or with the help of the supernatural: "an ailment is curable magically provided that it involves being dominated, a feeling of being taken over by a strange malign force..." [32]

In the districts of Ragusa and the Madonie whoever is 'diagnosed' with the frights is said to have haunted eyes. If inadequately 'cured', this psychological discomfort can become chronic and result in tension, spasms and globus hystericus and could also be the root cause of some symptoms of hydrophobia, anorexia, migraine, anxiety, insomnia, anguish and a sense of tightening of the throat [7].

In a 'magical' dimension, 'fright' is considered to be the cause of serious health risks. Its suddenness and unexpectedness could be due to many things. Its negative effects do not end once the fright is over but can persist to be unleashed later in a pathological reaction, even madness and sometimes death. Since childhood helminthiasis is also linked to fright, it is a very dangerous illness. According to popular belief, as soon as the child is born and before his first bath, it is opportune for his little hand to squash a fly or earthworm in the presence of a good healer who recites an appropriate prayer to render him immune to intestinal parasites. Another tradition was to put an earthworm in the hand of a newborn baby, wrapping it there until the worm died or until the baby was baptised, thereby protecting the child for the rest of his life from worms, and in turn would have been able to free others from parasites on condition that his mother took care to recite the following spell [33]:

'*Sennu paganu, tinni u vermu a manu:/ora lu 'mmazzu ca sugnu cristianu*'.

'Being pagan, I held the worm in hand: now I'll kill it because I'm a Christian'.

In the meantime, the healer would have had to make the sign of the cross on the child's stomach and give an oration originating from Acicatena (Catania) whose last two couplet say:

*'...lu jornu di Pasca/lu vermu ti casca'*.

'...on Easter Day, the worm will fall'.

From the data of a recent enquiry (September 2007), the practise of charming worms is still operative in Villarosa where it is carried out for three consecutive days – at dusk say some and, say others, just before dawn – in the following way: the parasite inflicted patient lies horizontally so that the healer casting the spell massages the stomach starting at the top. The massage and palpations allow the healer to verify if parasites are present, if they are single or in groups, and their position in the intestine. Furthermore, by means of these repetitive movements which require delicate contact with the patient, the healer can help relax the patient which is essential in this type of ritual healing.

Whoever has the power to cast a 'spell' begins the ritual with the sign of the cross and then does three small crosses (which means cutting out the evil) on the patient's stomach: one is at the top of the stomach and the other two on the sides. As he does these crosses (*fa i cruci*) he begins a 'prayer', also known as a 'tale', to charm the worms (*a-razzioni dâ cirmata dê vermi*) or more simply a propitiatory prayer (*a cirmata*). It can be sung softly or like a rhythmic lullaby which, together with the massage, helps the patient relax and become self-confident thus acquiring psychological wellbeing.

According to some sources, also from Villarosa, charming worms must be handed down from man to man, or woman to woman or from man to woman or vice versa. As we know, whoever learns the art of charmer cannot exercise it if his teacher is still living. The words of the 'charm' may be modified; even the tradition of a charmer waiting for his master to pass away before he starts practising is not always true. This variation is due to the fact that in the past Villarosa was home to various ethnic groups from neighbouring areas (eg. Sperlinga, Ganci, Alimena) who brought with them these changes to the original. Historically, Villarosa was essentially an agricultural village where, every June, reapers (*spicalora*) from neighbouring villages would arrive to help harvest the corn at Duke Notarbartolo's estate, which at the end of the nineteenth century became a mining area after the discovery of sulphur.

### "The saintly hand"

The interviewees often emphasised that worm charming was not an easy job: whoever does it must have faith. Among the charmers some seem blessed with the 'virtue of birth' a sort of 'magic power', a sign of greatness. Others possess the so-called 'saintly hand' which has been defined as a type of 'power' acquired by putting any type of earthworm, a cabbage caterpillar (*un virmuzzu*), a bait worm (a casièntula) or caterpillars of *erva di vermi *(genera *Verbascum*) in the palm of the hand which is then closed and bound while a spell is cast. The hand should remain closed until Good Friday. If, on that day when the hand is opened the worm is dead then that person has acquired the 'gift of the saintly hand'. In terms of healing, whoever is charmed by that person, may rest assured that they will be healed forever from worms even if they later suffer from a strong 'fright'.

A procedure has been described [12], similar to that at Villarosa: on Christmas night during the nativity, or on Easter Day during the blessing of the water, whoever wants to acquire the 'gift' should go to church with someone who knows which spell should be recited. Then, in May on the night of a full moon, he should go into the countryside, find a thistle caterpillar, rub it between his hands, then have his hands bound by the spell-sayer, and lastly have the remains of the caterpillar pushed in between his hands. Three days later, the hands unbound, the subject now has the power to heal others from worms.

#### Curses and prayers

During our enquiries at Villarosa, we obtained the prayer-curse which the healer recites while his hand is tracing the three small crosses on the stomach of the patient with worms:

'*Luni santu, Marti santu,/Mircuri santu, Juvi santu,/Venneri santu, Sabatu santu,/Pasqua e-Ppascuni/cadi lu virmi a-ffaccia-bbuccuni. Tagghju unu, tagghj-ddui,/tagghju lu vermi c'aviti vui,/Tagghju-ttri, tagghju quattru, tagghju çincu,/taggliu lu vermi di lu cintu,/tagghju sei, tagghiu lu vermi di lu feli,/tagghju setti, tagghju ottu, tagghju novi,/tagghju lu vermi di lu cori/tagghju deci, tagghju lu vermi ca ti reggi*'.

'Blessed Monday, Blessed Tuesday, Blessed Wednesday, Maundy Thursday, Good Friday, Blessed Saturday, Easter Sunday, Easter Monday, falls the worm face down. Cut one, cut two, cut the worm which thou has, cut three, cut four, cut five, cut the worm from the belly, cut six, cut the worm from the bile, cut seven, cut eight, cut nine, cut the worm from the heart, cut ten, cut the worm which has a hold on you'.

The words of the spell lead us to believe that the charm is total because it removes the worm from the belly, bile (which stands for the liver and gall bladder) and heart which means from wherever – so it removes the worm which 'has a hold' on that patient as though that person were possessed. Furthermore, the spell mentions the holy days of the week since according to popular tradition they have considerable therapeutic and 'miracle' value particularly against worms.

The practise of the ritual above is said to be still operative today around Sperlinga and Valguarnera as well as Castel di Lucio and Gangi even though the incantations differ in content but not objective. It is said that spells are also cast in Ispica, Acicatena, Prizzi, Partinico, San Biagio Platani, Noto, Tortorici and Mistretta.

Again according to popular tradition, the reason some children's noses itch is because the worms have reached there from the intestines invading the throat and lungs and giving rise to an irritable cough and so it is opportune to also cut away the worms in the 'chest' and in the 'heart' as cited in a spell originally from Mistretta: '*Tagghiu u vermu nta stu cori*' ('cut away the worm from this heart') and from another originally from Modica:*'Tagghiu stu viermu ntô mienzu u cori' *('cut away the worm which is in the centre of the heart').

It is reported that unusually stomach worms can climb up through the stomach and oesophagus and into the bronchi during sleep where they later cause abscesses or gangrene. This is alluded to in the following spell from Ragusa county:

'*Vermu, virmuni,/libera stu prumuni,/nun essiri fitenti,/pirchì si-ccurtu e-ppuzzulenti*'

'Worms, big worms, quit this lung, don't be so rank, because you are short and stinking'.

In Mistretta, there is an extremely 'complete' spell in that worms are cut out from every part of the child's body and at each stage a cross is made. Moreover, it imposes a caveat on the Madonna asking for glory in Heaven as a prize deserving to those who do good by helping sufferers [12].

'Vi salutu Bbedda Matri Maria/spusa di l'arma mia/e vogghiu-zzoccu m'aviti prumisu/La santa gloria rû Pararisu'

'I salute you beautiful Mother Mary/wife of my soul/and I want that promised me,/blessed glory in Heaven...'

The spell continues in the usual way (more or less similar to the one in Villarosa):

'*Tagghju unu, tagghju-ddui,/tagghju lu vermi c'aviti vui,/tagghiu-ttri, tagghiu quattro...*'

'Cut one, cut two,/cut the worm from your life,/cut three, cut four...'

And so on nominating various parts of the body in which worms are cut away:

*'nta stu nasu' *('from this nose'), *'nta stu cintu *('from this belly'), *'nta sti vesti' *('from these clothes'), *'nta stu cori' *('from this heart'), *'nta stu corpu' *('from this body'), '*e-ppi tutti li vii' *('from wherever the are').

At the end the healer recites:

'...*tagghiu novi, tagghiu deci/tagghiu u vermi a stu poviru 'nfilici*'.

'cut nine, cut ten,/cut the worm from this poor wretch'.

Then, he lists the saint days (see Villarosa spell above) to which are attributed significant therapeutic value (not only in Sicily).

In the spell above, simply listing the parts of the body in which worms may lurk testifies to the popular belief that parasites are ubiquitous which explains the commitment required to carry out the cuts. These must be numerous given the most unlikely places in which worms may lurk. The cuts in this and many other spells are repeated ten times because ten is the perfect number. The importance of the cuts lies in the fact that an expert can eliminate '*u vermu mastru' *('the master worm'), the one that keeps together all the others in a '*gghiommuru' *(string ball), that knot which, contorting in all directions, so torments little children.

With the following spell from Modica, four odd cuts are made (3, 5, 7, 9) to liberate a little girl who has worms 'in her heart':

- "*Unni vai, virmuzzu mancuni,/ca la picciridda mi murìa? – Tu chi 'un sapevi a priera mia,/ca la picciridda 'un ti murìa? "Tagghia-ttri, tagghia çincu,/tagghia setti, tagghia novi, tagghia stu vermi ch'è 'mmienzu lu cori:/Santissima Triniati, livaticci 'a malata/e lassàtila in libirtati".*

'Where are you going little wormy worm,/that this child is dying? – Didn't you know my little prayer,/that the child would not die? Cut three, cut five,/cut seven, cut nine, cut this worm that is in her heart,/Holiest Trinity, cut this worm from the sufferer/And leave her in peace.'

On more than one occasion, it has been said that worms can be found anywhere; their ubiquity is extraordinary and so too their ability to move around the various organs of the body amongst which the liver and lungs as can be seen in this spell from Naro. The theme of the sea runs throughout, highlighting the importance of this element in medico-magical and propitiatory rituals in which its great virtue is its purifying force:

'.... *Stàiu viniennu di dabbanna u mari,/haiu 'ncuntratu lu granni virmuni/stava iennu a manciari ficatu e-ppurmuni./Ppi-ll'amuri di lu Signori, iettati buccuni,/nun 'nquitari li criaturi*!'

'...... I come from the other side of the sea,/I've met the great worm/which was eating liver and lungs./For the love of God, throw yourself down,/And don't alarm the poor children!'

Intestinal parasites can inflict anyone and according to ancient belief even Saints as pointed out by some spells from the provinces of Agrigento and Palermo. In them, Saint Peter 'has a worm which is *u granciulìa *(tickling him)', Saint Anastasia '*havi lu vermu di la vìrmaria' *('has a worm from the worms nest') and Saint Rosalia's '*si smuvìu a virminìa' *('worms moved').

This spell from the province of Messina describes the suffering of Saint Laura:

"*Santa Laura chiancìa e supra lu munti sidìa./U-Ddivinu Maistru ca passava ri-dda, cci dissi:/Santa Laura pirchì chianci?/e la Santa arrispunnìu:/Haiu u vermu ca mi mancia viva./U Maistru ci rissi allura: Ricci la mè-rrazziuni!/E Laura arrispunnìu: Signuri, iu n'à sapìa/*

*U Signori dissi allura: ricci-ccû-mmia:/Tagghiu unu e-ttagghiu-ddui, intra u mè ventri nun vinari-cchiùi,/vermi, virmuni, mori ntâ st'agnuni!*".

'Saint Laura cried and sat on a mountain./The Divine Master who was passing by said:/Saint Laura, why are you crying?/and the Saint replied:/I've a worm that's eating me alive./So, the Master said: Recite my prayer!/And Laura replied: Lord, I do not know it!/So the Lord said: say after me:/Cut one, cut two, never return to my stomach,/worm, great worm, die in this corner!'

Even *a Maronna *(the Madonna, so says popular belief, 'cuts out' worms and advises prayers as these two worm spells from Sciacca and Corleone evidence: the Madonna may be invoked to strengthen the 'mortal' hand of the healer. The divine hand is invisible which with supernatural force strengthens the 'healer'. So, the conviction that the healer's practises against helminthiasis are valid is supported by a present and operating force of God during the rituals as explicitly highlighted in the following spell from the province of Agrigento:

'*Ddiu è assai-cchiù putenti ri l'omu,/chistu è-cchiù putenti di la terra: è-Ddiu ccû la so' putenza,/e no tu, mavàra, ccû la to' scienza*'.

'God is much stronger than man/who is the strongest on the earth: And God with his strength,/and not you, old witch, with your art'.

In many municipalities in the province of Messina (Acquedolci, Caronia, Cesarò, San Fratello, Galati Mamertino) and in their surrounding areas, this research was able to highlight through dialogue and interview how in the Nebrodi Mountains until a decade ago – and in some cases even today – people resorted to firesinging (*pircantari*) even for curing ailments other than helminthiasis.

Various sources affirmed that sciatica, cephalea and St. Anthony's fire or herpes (*dugi di San Antoni*) were cast out by spells. The latter is still cast out today in Belpasso on the slopes of Etna. In this case, the 'healer' casts his spell by praying and spitting on the infected area in the belief that his saliva has healing properties.

In the Nebrodi mountains, and this is still believed today, newborn babies and very small children can suffocate if the parasites climb the respiratory tract. Whenever mothers fear this is the case, they *pircantunu i viermi-ccû-ll'iègg e u filu dû rrucchièddu e-ttagghjanu u scantu *'curse' the worms with garlic and a thread and 'cut the fright' by using a magical practise. Sometimes – as happened more often in the past in the days of paraffin lamps – they dip a finger in the paraffin rubbing it on the throat, stomach, armpits, soles and behind the ears so that its intense odour crushes the parasites.

From a source in Caronia we learned that you can measure whether a person '*havi u scantu' *(has the 'fright') with a '*ccû-nfilu ri cuttùni*' cotton thread by standing the person '*âddritta e-cchê razza aperti*' (straight with arms held out by their side). If the person is afflicted the fingertip to fingertip distance will be greater than their height. That being the case, the 'healer' who carries out the 'liberating ritual' (usually a woman) pronounces the words of the 'spell' while folding the cotton upon itself to a length of 6–7 cm cutting the bunch at its two ends to obtain many segments with which to arrange small crosses all over the body of the resting 'patient'. At this point in the ritual, the 'healer' boils down a glass of wine to a third and adds three red hot embers which the 'patient' must then drink toasting the 'fright'. This ritual must be repeated on three consecutive days or three times in one day: in the morning, at dusk and again the following morning before the sun is high because it is believed the sun excites the illness stimulating the movement of the parasites.

A source in San Fratello spoke about a practise which was quite similar to Caronia's regarding the casting of spells for children's worms but with a few variations. The ritual is carried out with garlic, oil, water and a thread. Firstly, the garlic is chopped up and put in a glass of water. Next, the 'healer' anoints her hand with a little oil and, after making the sign of the cross, performs a circular massage on the child's stomach affirming that the child really has worms, after which the following *historiola *(prayer or 'story') is recited three times whose magical power lies in the evocativeness of the words. In this very well-known prayer the 'story' is of the holy week up to the moment of the Resurrection:

'*Luni santu, marti santu, mercuri santu,/jovi santu, venneri santu,/sabatu santu, duminica di Pasqua,/u vermu n terra casca*'.

'Holy Monday, Holy Tuesday, Holy Wednesday,/Maundy Thursday, Good Friday, Holy Saturday, Easter Sunday,/the worm falls to earth'.

Following this, the 'healer' cuts a piece of thread with her left hand, makes the child stretch out its arms and hands at its sides and then measures the overall span. Another thread is cut to measure the child from head to foot. Then both threads are taken, knotted together and wound around the index finger and thumb of her own hand. The wound thread represents the entanglement of the worms. They are then removed from the hand, cut up (as though cutting the worms) and placed with one finger into the water with garlic. If a thread immersed in the water remains straight there are no worms; if they curl up and intertwist this indicates how many worms are present. Next, the 'healer' turns to the mother (Maria) of the child (Giovanni) and asks her: *'Maria, cchi-ttagghju?' *('Maria, what are you cutting?') to which Maria replies: 'The worms'. Then, the 'healer' says '*Allura, tagghjàmucci i viermi a-Ggiuvanni*' ('Well then, lets cut Giovanni's worms') and makes the child drink on two-three consecutive days the water with the garlic (thread removed). The 'healer's' cutting with the left hand is typical of several magic procedures since 'Magic likes to do the opposite of religion and social life' [12]. The thread cutting ritual is comparable to that carried out during child helminthiasis therapy reported a 'healer' from Sagama (Sardinia), according to whom a linen thread must be used which is as long as the child is tall and then cut into seven equal parts. Next, water is poured into a glass or onto a plate, some prayers are recited amongst which the Credo, and finally this formula:

'Holy Monday, Holy Tuesday, Holy Wednesday, Maundy Thursday, Good Friday, Holy Saturday, Easter Sunday, wherever the worms are, they fall' [34].

Every time a day of the week is cited, a piece of linen thread is dropped into the water. As in the case above, if the child is really suffering from helminthiasis, the pieces of linen will twist together; if they don't, there are no parasites. Should the illness be verified, the linen threads should be cut up and burnt. If the 'patient' is too young, the thread ash should be put on his navel or otherwise, thrown away. After treatment, the healer asserts that the child will heal soon. Other healers we contacted said they use the same therapy with some small variations. In any case, the prayer must remain absolutely secret and the ashes must be dissolved in some liquid which the patient must drink. Furthermore, the cure should be carried out at the end of the lunar cycle; at the end of the ritual, the thread and water are flushed away [34].

In some areas of the provinces of Ragusa and Siracusa they also use magic to *calari a virmina *(heal children's worms); it consists of a special abdominal massage, a sprinkling with oil, and the recital of particular prayers. In the Etna area, it is well-known that following a 'fright' the worms are no longer *cheti ntê vuredda *(tranquil in the intestine), all curled up together or like a 'doughnut', but are wriggling and jumping with painful consequences for the children.

It has already been said that in the Belpasso area, *cirmata *(casting spells) still goes on today as it does in other municipalities of Catania and Enna, and that it is known as the *vermu tagghiarinu *o *tagghiarina *(tape worm). The 'spell' that was cast, but which continues to be cast in some areas of Etna, is the following as told us recently:

*'U Luni santu*, *l'addevu è spantu,/lu Marti santu, lu vermu è ntô sô mantu,/lu Mercuri santu, lu vermu è ntâ lu cantu,/lu Jovi santu, si fici u pircantu,/lu Vènniri santu è-ttuttu 'n turmentu,/lu Sàbbatu santu, fù comu d'incantu,/la Dumìnica di Pasqua u vermu, cumprìtu, casca*.'

'Holy Monday, the newborn child is frightened,/Holy Tuesday, the worm is in his guise,/Holy Wednesday, the worm is in his corner,/Maundy Thursday, the spell is cast,/Good Friday is torture,/Holy Saturday is delightful,/and on Easter Sunday the worm falls whole'.

From the text of this spell, there is first a description of the escalation of the illness and then, thanks to supernatural intervention, its gradual remission. Initially, the young patient is frightened and in the days following the 'healer' identifies where the parasites are – at first in their 'cloak' that is curled up in a ball and then on one side of the stomach. At this point, after the 'spell' and suffering of strong pain, recovery begins as though by magic, and, finally, the worm is completely ejected. This prayer highlights more revealingly than others the healer's ability while massaging the patient's stomach to perceive whether or not the worms are intertwined or if they are at the centre or sides of the intestines.

In another recently discovered popular spell against parasites, the healer addresses: the worm as a challenge, the small patient inviting him to move his bowels, and finally the patient's mother telling her that her son would be renewed and no longer frightened. The spell mentions the patient's mother again who, at the end of the ritual, observes incredulously the healing obtained and sings with joy:

'*Santu Luni, unni vai-ccû stu cipìgghiu?/Santu Marti, unni vai ca ju ti pigghju/santu Mercuri, unni vai ca ju ti tagghju/Santi Jovi, li toi visceri rimovi/ca stu figghju si rinnova/Vènniri e Sabatu santu, u tò figghju n'è-cchiu spantu/Duminca santa, la matri varda e-ccanta*'.

'Holy Monday, where are you going with that frown? (to the worm)/Holy Tuesday, where are you going because I'm going to catch you?/Holy Wednesday, where are you going because I'm going to cut you?/Maundy Thursday, move your bowels/So this son is renewed/Good Friday and Holy Saturday, your son is no longer frightened,/Easter Sunday the mother watches and sings'.

Finally, a spell fragment from near Palermo referring to the final liberating phase of the healing of a woman (or child)

'*... figghja mia tallibbirtasti/di li viermi e-ddi li mprasti'.*

'...my son, you are free/from worms and suffering.'

### "Plants which cure worms"

Worm infections can be of various types: one is teniasis involving the tapeworm or solitary worm, an endoparasite of the platelminti (flat worm) family which live in the intestines of both man and animals. This parasite is very common and can live as long as 25 years if no surgical or pharmacological therapy is practised. There are many tapeworm species: among the most common which infect man are *Tenia solium *whose intermediate hosts are wild boar and pigs, and *T. saginata *whose intermediate hosts are prevalently cattle. In the industrialised countries the incidence of this parasite is much lower than in the past, given that health and hygiene norms are considerably higher.

As regards the double pathological-emotive aetiology which would give rise to an attack by intestinal parasites and the two therapeutic remedies of 'spells' and antihelminthiasis plants, a source in the municipality of Sant'Alfio described a cure for tapeworms using the male-fern (*Dryopteris filix-mas*, *fìlici masculina*) which was well-known even in neighbouring districts.

It should be stated beforehand that the information reported goes back to an episode from about fifty years ago when, in that and many other areas, childhood worms were very common because farmers' children and their playmates often ate raw or under-cooked pork (sausages, non-aged salami). The remedy describes (the witness had to take it himself from his own father, a gynaecologist) a glass of a very dense herbal decoction with a foul taste obtained by long-boiling the male-fern [35]. The drug from that species' rhizome is very toxic so its use as an antianthelmintic should be under strict medical supervision [23]. Six hours after taking the 'medication' the witness recalls very strong stomach cramps due to the worm's 'contracting and writhing'. Then, a chamber pot (*càntaru *or *cantru*) was used to check that the worm had been totally expelled including the head: if the head remains in the bowels it can reproduce. Tape worms look like a long tape in numerous segments (proglotti) of 1–1.5 cm with a head equipped with suckers and hooks so they secure themselves to the intestine walls. The remedies prescribed also vary with the gravity of the symptoms. However, generally speaking different herbs, leaves, tree or shrub twigs, various tubers, heated oil and wine are used.

Some women who are not necessarily expert healers and occasionally incapable of carrying out rituals or spells but who are usually related to the child, use coffee either ground or in beans. For ground coffee, the dose is a teaspoonful which the child must smell on the morning of the day when he will be treated for worms. For bean coffee, the beans need to be crushed quite finely to release their stimulating and therapeutic effect. It would seem that it is the released aroma which aids the worm's expulsion. In a town in the foothills of Etna, the following spell was used to free a young girl from worms:

"*Cafè, cafiùzzu, lévaci stu virmuzzu/, lévalu in nomi di Maria/accussì nun cutturìa-cchiù a figgliuzza mia.*"

'Coffee, little coffee, get rid of this little worm,/get rid of it in the name of Maria,/so that she is no longer troubled by it'.

The plants commonly used against the 'frights' and the consequent helminthiasis are still those recognised as having antianthelmintic properties: onion (*Allium cepa*, *cipudda*) and garlic (*Allium sativum*, *àgghiu*) [35-37]. In the province of Ragusa, around Modica and Monterosso Almo, the tradition continues to cure colic and 'fascinate parasites' in children by making them swallow several cloves of garlic or putting them in their cots and furthermore by rubbing half a clove on their backs or under their noses. Another solution is to crush a couple of cloves and push them into the child's rectum together with a drop or two of the garlic liquid. The best remedy of all is for the child to wear a necklace or crown of bruised garlic cloves since they are attributed with exceptional anti-worm powers and notable apotropaic qualities [38]. Before casting the spell, the healer asks the mother of the small patient for thirteen cloves, or less if they are not available, but they must be an odd number: eleven, nine, seven or even five, but no less since the necklace wouldn't be able to be made. This shows that, although some plants really do possess antiworm properties, the 'magic' factor also needs to be considered. Garlic which is the best of the antianthelmintics, is used as a necklace because its smell 'stuns the worms', but it also necessary that there be an odd number of cloves for the 'magic' factor.

The necklace is made by piercing the cloves and threading them onto a strong piece of cotton which is knotted around the child's neck and worn for the three days of the cure. It must be made by whoever casts the spell so that should no-one be available, a necklace made by the child's mother would have to be re-made. As soon as the clove necklace is knotted in place the healer recites a prayer. The one we recorded in an area of Eastern Sicily is the following, in which Saint Agata, the patron saint of Catania, cries disconsolately:

'*Sant'Aituzza assai làcrimi chianceva, ..../Passau lu Signuri e-cci rissi: – Aituzza, pirchì sta chiancennu? – Pirchì vinni lu vermu mastru e si sta manciannu la me figghiuzza*./

*Vermu unu, vermu-ddui, vermu-ttri, vermu quattru, vermu çincu, vermu sei, vermu setti, vermu ottu, vermu novi, casca stu vermu di lu cori *'

'Saint Agata cries many tears..../The Lord passed by and said: – Agata, why are you crying? – Because the 'Master Worm' is come and is eating my little daughter!/Worm one, worm two, worm three, worm four, worm five, worm six, worm seven, worm eight, worm nine falls thus from the heart.'

These garlic 'therapies' were very widespread particularly in the past and they allowed the worms to be 'fascinated' which then descended (*scinnivanu*) the intestines and were expelled (*calata rê viermi*). Even today, steeping three cloves in wine or oil for a night (*calata rê viermi*) and the morning after making the little 'frightened' child drink the 'potion' is still practised in some areas.

Another 'cure' (discovered between Petralia Soprana and Gangi) consists of making the child smell the garlic (*çiauriari l'agghiu*) or in placing a glass with a garlic rubbed rim on his navel (suitable also for adults), or doing a stomach massage with something oily (usually olive oil) all the while accompanied by the 'charm', the typical spell recited during the manipulation.

In the above mentioned municipality of Villarosa today, getting rid of worms requires a peeled garlic clove placed on a plate or table. To one side, half a centimetre of oil should be put in a small cup or sufficient to wet the thumb. The rite starts with the sign of the cross followed by making the patient smell the garlic and then, with the thumb wetted in the oil, the 'charm' begins by making three small crosses on the patient's stomach while reciting the days of the week. The 'healer does not necessarily have to have a 'sainted hand': what's important is that he knows the prayer well, and that he can sign the crosses with his thumb by making circular movements from the top of the stomach to the sides and bottom until the worm is expelled.

At this point in our dissertation, it would be opportune to parallel Sicilian therapy with those in Sardinia. As regards the occurrence of worms (*is bremis *– Sardinian for various forms of intestinal helminthiasis, except tapeworms-, it is generally reported [16]) as being associated with food and that those most prone are children. The most usual therapy provides for the use of garlic in different ways and principally pulped, diluted in a glass of water, and given to drink to the child patient or administered by enema. The garlic mixture is defined by nearly all the 'healers' as *s'aqua de s'allu *(the water of health). Garlic can be crushed or cooked in milk, mixed with coffee or wine, applied directly to the anus or wrapped in a consecrated wafer and swallowed whole [16]. A Sardinian lady suggested it was also beneficial to just smell the garlic because as soon as the worms perceive the odour they tend to exit the anus.

In Sardinia, there are other therapies although less widespread which consist in making the patient drink a decoction of two herbs il *pisus de santu Franciscu *e l'*erba de su zuddu *and giving him a chamomile enema. A decoction of this last herb given every now and again completely healed the son of another female source who had been close to death from parasitosis ever since he was a small child. According to another witness, worms 'bite' more after coffee. One day, this witness had eaten fresh chickpeas and, following them with a coffee noted that the worms no longer bothered him. Repeating the same experiment the next day – a lot of fresh chickpeas followed by coffee – and not feeling any pain at all, he noticed that later on he defecated four enormous worms. He therefore deduced that fresh chickpeas which contain a lot of salt had cured him of his illness [16].

Apart from the above-mentioned therapies, the patient could have: breathed in the vapour of boiling milk which would have helped him vomit the worms; chewed cheese keeping it in the mouth for a while, then spat it out and drank a glass of wine. Attracted by the wine, the worms would have been killed by it and then defecated. Another therapy consisted in applying kerosene to the anus or drinking bitter drinks based on lemon or pomegranate which agitated the worms – we found the expression '*li scinizzada' *which means that a drink 'agitates' the worms [16].

A female informant said it was sufficient to sing a song (unidentified) and according to another the *brebus *had to be recited which are the ritual words closely analogous to *u cirmu *(the charm) carried out in Sicily. The brebus '...describes magic formulae in general,...which were used not only for the 'frights' and the 'evil eye', but also in a large number of real situations in Sardinian agricultural life. Managing the formula was the prerogative of the user or holder alone; it could not be passed on reliably if not to a younger person who could only then use it successfully after the death of whomever had passed it to him. The formula was only made known to whomsoever needed it. The revelation of its contents for other uses could bring about a loss of effectiveness' [16].

Returning to tapeworm therapies (in sardinian dialect *su bremi solitariu *and also *is chirrus famius *or *is chirrus papadoris*), a full cup of milk had to be put in front of the patient's mouth. Since the tapeworm is greedy for milk, it would have come out to drink. At that point the patient would have had to be ready to grab the worm and extract it decisively. Even after a full meal, the patient had to drink a lot of milk. To be sure of expulsion, it would have been helpful to eat bread and onions, or else a lot of garlic. Furthermore, the writer [16] reports that during WWII, his brother had worms and that the State Doctor (*datturi de gruvennu*) who looked after the poor advised him to eat a lot of raw garlic. Following this suggestion, the worm was extracted and taken away by the Doctor in a bottle.

On the subject of extracting intestinal parasites from the mouth, this writer remembers that as a child, one of her elementary school assistants said that one evening one of her young relatives had suffered from stomach pains and a troublesome feeling of nausea. Then, she had felt breathless, sweated, coughed with a strong tightness in the throat *'comu s'avissi accupari-ppi-ssùbbitu*' 'as though she might have suffocated from one moment to another'. As the symptoms persisted, the mother intuited the nature of the malaise, made her open her mouth and stuck two fingers down her throat '*ficcannucci ddui ita ntê cannarozza' *extracting two worms that were on their way up *'chi stavanu acchianannu*', to the shock of those present.

All the ritual therapies for curing a specific illness utilise a general format which is then modified according to the knowledge and emotional 'involvement' of the healer who begins each ceremony by blessing the patient with the sign of the cross and reciting three catholic prayers: the Ave Maria, Our Father and Glory to the Father. Each prayer is repeated three times (although Glory to the Father is often not said). Afterwards, a prayer specific to the ailment is recited either three or nine times depending on the healer. The 'ailment' prayer has a common format. During the ritual, the ailment is firstly presented to a higher entity (the Holy Trinity, a Goddess like the Madonna, or a natural symbol like a rainbow). Then, Christ, the Saints or other male spirits are invoked who finally suggest a cure. This procedure is often followed by a rhetorical question directed at the ailment which the healer can decide to ask at the end of the ceremony together with another benediction or the sign of the cross accompanied by the Credo [18].

In many areas of Sicily, above all in the past and in addition to the magical practise of casting a spell *(li paroli di lu scungiuru)*, they used to give the child another 'scare cup'(*u cuppitieddu dû scantu*) in order to cut out the 'fright' or 'frightened blood' (*tagghiari u scantu *or *u sangu scantatu*) to eliminate any parasites – a pharmaceutical vermicide made from a small quantity of santonin (*santunina *or *santantunina*) and jam and placed in a glass or terracotta bowl also known as the 'worm cup' (*cuppitieddu *or *cuppiceddu di vermi*). Santonin is the principal active ingredient (but toxic, especially for the kidneys and nervous system) found in the flower tips of marine absinth (*Artemisia cretacea*, *assìnziu-serìfiu*). The ingredients of this electuary probably did not originate from Sicily given the absence of marine absinth on the island [19].

The custom of preparing the 'cup' is still in use today even in the countryside around Ragusa and Palermo; to make the anti-worm mixture the right kind of vermicidal herbs are collected, as confirmed by an old lady healer, to prepare the 'mixture' (*l*'*ammiscùgghiu*) to which jam, cream, honey, syrup or other sweeteners are added to render the medicine more palatable.

In Ragusa province, an apotropaic custom was the so-called 'collection of worms' (*cugghiuta dê viermi*), a type of prayer to curse the appearance of worms and which generally was performed during family reunions on Holy Saturday. To get rid of the worms, the mothers would tickle the child's navel with a thin parsley stalk. In that area, furthermore, another remedy for infant helminthiasis was the taking of 'worm seeds' (*Delphinium staphysagria*, *simienza rê viermi*), a seaweed (*Corallina officinalis*, coral moss) from coastal areas like Punta Braccetto, Punta Secca, Capo Scalambri, which are characterised by corals. This seaweed which contains vermicide [39] is inedible because it is calcareous and unpalatable so it is ground up. This seaweed is commonly dried and sold by the roadside and was used in a particular way. After having been washed and ground, these 'filaments' were used for 'salads' dressed with oil and lemon, or made into balls or fritters (*pulpitteddi e-ffritteddi*) with breadcrumbs or flour, quickly fried and served with honey and so eaten by children and adults.

As regards the pharmaceutical use of Sicilian seaweed [40] as a vermicide (including *Jania rubens *(*Corallinaceae*) [41]) the following should also be mentioned: *Alsidium helminthochorton*, *Sargassum vulgare*, *Digenea simplex *e *Ulva sp*.

In the Modica area, *simienza di viermi *(a.k.a. *cabbarasi*) is also well known, as its seeds are used in infusions because of its vermicide and laxative properties. Other well-known 'worm herbs', as the investigation confirms, are used as vermicides in folk medicine particularly in the territories of Caltanissetta and Caltagirone: great St. John's wort (*Hypericum perforatum*, *piricò*) [[26] :326 n IV] is described as a vermicide whose flower tips are generally used for visceral inflammation [21]. The Tree of Heaven (*Ailanthus altissima*; *summaccu arbòriu*) which has antidysenteric properties [21] and Spanish origanum (*Coridothymus capitatus*; *tamareddu*) which are popular medicines uses flower tips for vermifuge and antiseptic properties [23].

In the Caltagirone area, near the Borgo di San Pietro (St. Peter's Wood) and surrounding areas the custom of using herbs is highlighted by this proverb: 'Every herb has its own virtue' *(Ogni erba havi a so' virtue)*. As a vermicide, local folk use various species of fern (*Athyrium filix-foemina*, lady fern, *filicia*) whose rhizome is used against oxyuriasis and the below cited male fern whose rhizome's [21] main active ingredient is toxic [23] in high doses and has been the cause of occasional poisonings in the area. Among other species of note in the Caltagirone area, the vermicidal properties of some were brought to our attention by local collectors (found even in the literature [42]): green purslane (*Portulaca oleracea*, *erva pucciddana*), wild carrot root (*Daucus carota*;*frastunaca*), 'love in the mist' seeds (*Nigella damascena*; *simenza dâ fraulara *[23]), corncockle (*Agrostemma githago*; *aìna picciridda *[23]) whose seeds are no longer in use because they are toxic [21], American wormseed (*Chenopodium ambrosioides; tè sicilianu*) of which the fruit, seeds and flower tops (containing ascaridol, attributed vermicide) are used.

In some parts of Sicily, the tansy (*Tanacetum vulgare erva di vermi *or *erva atanasìa*) was used hence the folk saying: 'If the worm is corroding your insides, you have to taken tansy'(*Si lu vermu d'intra ti sfirrìa/t'ha pigghjari l'erva-atanasìa*). The plant is said to have antihelminthiasis properties against pinworms and ascarids [23].

Some old healers we contacted referred to 'side effects' of the 'frights' like jaundice (yellow or little yellow; in Ragusa saffron) which they cured with herbs and was believed, above all in the past, to arise after a particularly big fright. The 'little yellow', according to some data from Caltanissetta, is cured by Field Eryngo (*Eryngium campestre, panicaudu*).

### "Plants used against helminthiasis"

Before evaluating the properties of some 'vermicidal' plants as declared by some of our informants, it is necessary to make a clarification. Often, either because of inadequate botanical knowledge or out of the habit of using plants whose officinal properties have been handed down orally without evidence of their real efficacy, 'vermicidal' plants included both those which were really effective (marine absinth) as well as those which were effective against the side effects of worms such as gastric swelling, intestinal disorder, halitosis and others (with properties like anti-flatulence, digestive, cholagogueic, antispasmic and aromatic).

As regards vermicidal plants, some are known as 'spell plants' (*ervi di lu scunciùru*) whose therapeutic effect is due not only to their main active ingredient but also to the value attributed to those plants by popular belief. It has been said that: "Sometimes it is not God or a Saint whose help is asked for to fight worms (but) a medicinal plant" [12]. In the following spell which comes from Prizzi, rosemary, which has long been known to have multiple officinal properties, is invoked:

(*Ti scunciuru malu natu,/ti nn'a-gghiri unni sì natu:/ca Ddiu nascìu, Ddiu arriviscìu,/ppi sarvari a-nnui; rrosamarina, chi Ddiu ti binidici, dunami la virtù/chi Ddiu ti fici*).

'I implore you wretched thing,/you have to go back to where you were born:/because God was born and God was resurrected/to save us; rosemary, may God bless you, give me the virtues/which God gave you'.

The healer who cast this spell addressed a real prayer to the rosemary asking it, in the name of God, to give up its God-given powers: its antiseptic properties. Permeating with the powers of the healer, these properties succeeded in finding and beating the parasites in the child. Perhaps of old, before or after having cast the spell, a rosemary decoction was given to the suffering child or some sprigs were burnt to fumigate his room. This latter was probably the preferred tactic both because of its effectiveness and because it was more suitable for babies.

Other vermicide species were used in stomach poultices (*stumacali*) since they possessed special organoleptic characteristics which, according to our sources, made them unbearable to worms amongst which was rue (*Ruta chalepensis*, *aruta*) containing glands which secreted strongly aromatic essences used in the Ragusa area for its capacity to ward away worms. The same was found in some hamlets around Petralia Soprana where, whoever has children, will do anything to get hold of some rue which could be sniffed by the small patient or macerated for 24 hours in a glass of water and then given in moderate quantities in appropriate doses.

Also in the area of Castellana Sicula, popular tradition attributes rue with vermicide properties which are particularly appropriate for children; for this reason, mothers usually placed a sprig of rue under the child's pillow or mattress.

An elderly herb collector from Polizzi Generosa commented that 'when helminthiasis symptoms are very uncomfortable' (*contru i viermi forti*) mint may be used which is carminative and aids digestive problems. The patient must chew the leaves; mint can also be given as an infusion after macerating in hot water. Others in the area said they generally used the juices of cooked leaves (or a decoction) of snow thistle (*Sonchus oleraceus*; *cardedda*), a plant with tonic and cholagogic properties. The potion should be taken by the glassful which is known as 'the glass of frights' *(u-bbicchieri rû scantu)*.

According to a hundred-year-old herb collector from Calcarelli (a hamlet of Castellana Sicula) gastric pains (*u scantu e u rulur'i panza*) can be soothed with an infusion of water and bay leaves *(Laurus nobilis*) due to the eupeptic and carminative properties of the plant. The infusion, which is boiled in half a litre of water and the appropriate quantity of bay leaves is known as *addàuru vuddutu *(boiled bay leaves) or *canarinu *(canary).

Another vermicide species used in the past was the so-called 'white herb' [26] or wormwood tree (*Artemisia arborescens*, *erva-ianca*) whose flower tips were used (both in the Madonie Mountains and in Caltagirone) in folk medicine for 'ailments of the stomach'. In the countryside around Ganci, according to local sources, 'soothing worms' (*ppi calmari i viermi*) was done by placing a sprig of 'white herb' on the chest (*ntô piattu*) of the patient or alternatively the juice of the plant was squeezed (*si smuncìva u sucu*), mixed with a few drops of olive oil and then given to the child to sip with his nose held (*ntuppànnucci u nasu*). The 'juice' of 'white herb' could also be mixed with a spoonful of human or goat milk.

As in other above-mentioned areas, in Santo Pietro di Caltagirone both rue and 'white herb' are used to fight worms. The local folk collect enough rue leaves to extract three drops of juice (*ttri stìzzi ri sucu*) which dose must be taken once or twice consecutively. In that area, rue is esteemed for a number of other therapeutic properties as this proverb testifies: "*A marva ti sarva e a-rruta ppi ogni autru mali*" (mallow saves you and rue is for every other ailment).'White herb' is used by boiling it, then filtering the cooking juices and administering by enema.

Above all in the past, when children were suffering from painful and swollen stomachs, a courgette was peeled and put on the painful area, whereas to 'remove' worms flakes of homemade soap were placed on the stomach [17].

In Giarratana and in general in the province of Ragusa, it seems that in the past *a sudda sarvaggia *o *erva-ri-pittrògghiu *(pitch lover, so-called because of the odour of pitch) was used as an oil substitute in 'curing' infant helminthiasis. The freshly gathered flowers were held to the suffering child's nose.

In Alcara Li Fusi and Militello Rosmarino the fresh leaves of wild chamomile (*Achillea ligustica*, *canfuridda*) are inhaled as a vermicide, or placed in a cloth bag and put under the child's pillow. This practise is echoed in a popular belief that worms will spontaneously climb up the alimentary canal but once they scent the plant they would reverse their path and be expelled [3]. The officinal use of this plant has also been noted in the rural community of Mezzojuso [43].

In the territories of Monterosso Almo e Giarratana, children suffering from intestinal parasites (*picciriddi cchê viermi*) were made to drink an infusion of the flower tips of the pitcher plant (*Calamintha nepeta; spicuna rî nièbbita *or *nipitedda*) soaked in boiled water which was believed to relieve the symptoms of helminthiasis. Even today, the pitcher plant is used as a peptic to calm stomach pains and other gastric problems. Their flowers can be crumbled and repeatedly sniffed which apparently soothed abdominal colic. Furthermore, after being soaked at length in hot water, the pitcher plant, according to a local source, was given to eat to whining children *(picciriddi siddusi)*. It is also reported that 'its juice drunk with wine knocks out worms [25]. In the territory of Mistretta, the crumbled leaves of the pitcher plant (*niputedda*) are fried in olive oil and garlic; then they are rubbed into the navel to get rid of worms [44].

Even peach can be a cure-all: as an anti-helmintic both the peel and the '..leaves are excellent vermicides..' [30].

A procedure to cure 'fright worms' is practised by a 'healer' from Cefalà Diana who, reciting a prayer, used a small vine (*Vitis vinifera; sarmentu*) shoot enlarged at one end (to simulate a head) and with two small holes as 'eyes' to make the sign of the cross on the mouth, forehead and shoulders of the patient. Then, still reciting the prayer, pressed the vine shoot onto the mouth and stomach (*supra a vucca e supra u stomacu*) and then threw it into a glass of water while reciting the Lord's Prayer with the patient. If the shoot opened in the enlarged area as though it were a mouth (*comu si fussi na vucca*), this meant that the patient had worms and had had the 'frights'. At the end of the ritual, the patient drank a little from the glass; what remained was thrown out of the house so no-one else present could catch the 'fright'. In this ritual, the vine shoot represents the worm and also the force to eradicate it. However, it is the ritual which confers value to the power of the shoot/worm (*sarmentu/vermu*) which has been absorbed by the water. The signs of the cross have an exorcising significance, but are also a type of benediction [31]. Quite a similar procedure for curing worms in young patients was recently discovered in the province of Catania in an Etna village which once had extensive vineyards. The healer's spell refers to a swollen vine shoot which after immersion in water opened its 'mouth' (*ddoppu essiri statu misu ammoddu*, *avìa rapùtu a vucca*) signifying the presence of the ailment:

(*Viti, viticedda/la me figghia nun è-bbedda/nun è-bbedda, picchì è-mmalata/ogni notti-cci fazzu a nuttata.*

Viti, viticedda/nta l'acqua, rapisti a vucchicedda/pirchì a me figghia è-mmalatedda/Ora facemu a priiéredda/ppi falla turnari bbedda-bbedda.

*Patri, Ave e-Ggloria dissi la fighiaredda/e-vippi l'acqua unni era a viticedda/U scantu passau e u jornu agghiunnau*).

'Vine, little vine/my daughter is not beautiful/she isn't beautiful because she is ill/every night I am by her side.

Vine, little vine,/you opened your small 'mouth' in the water/because my daughter is ailing/now lets say a little prayer/to make her beautiful again.

Our Father, Ave Maria and Glory to the Father says the young daughter/and drinks the water where the little vine was/the 'fright' has passed and the light of day has come' (ie. metaphorically the healing).

In some areas, another remedy against the 'fright' that, if very strong, they thought could thicken the blood (*siccari lu sangu*) was made with an infusion of dog rose (*Rosa canina*, *rrosa sarvaggia*) petals (tonic and analeptic properties) macerated in wine. With the fruit of this plant, the rose hip which contains small seeds, a laxative herbal tea was prepared; the galls which grow on the branches are both tonic and astringent and can make an antispasmic decoction. Regarding the officinal properties of rose hip (*cinorruodu*), [27] reports: "...from time to time you can see threadlike excrescences [Rose bedeguar gall, Robin's pincushion gall, or Moss gall] above the stems produced by the stings of the Gall Wasp (*Cynips rosae*) which ...are used as a vermifuge ingredient in pills [usually rather large to administer huge doses of unpleasant tasting medicines] and the common folk prepare a winey infusion with it to cure the consequences of 'terror"'. It should be made clear that, in this context, the term 'terror' above should be read as a synonym of 'frights' which has been used thus far to mean a condition of 'great emotional discomfort' which is thought to be responsible for both infant helminthiasis as well as numerous other psychophysical malaises.

The use of medicinal plants is often indispensable during healing ceremonies, but the spells, prayers and invocations of sacred beings also play a fundamental role. Often, during the ritual, the healer submissively recites or sings delicate prayers dedicating himself to other activities like gently massaging the patient or waving herbs about or rubbing them on the painful area. A good example of this came out of some research in Casalvecchio Siculo. On that occasion broadleaved pepperweed or 'sciatic herb' (*Lepidium latifolium*) or the 'small flowered willow-weed' (*Epilobium parviflorum*, *erva sciàtica*) was being used officinally and in magic rituals. In that area and roundabout, the plant which is used to treat neuralgia of the sciatic nerve, was being used in a therapeutic ritual during which the 'healer' recited a particular prayer (*prigantozzu*) and rubbed the plant delicately over the painful area [6].

It should be highlighted that the cadence of the spells and songs plays an important role in the 'healing' because it has beneficial effects on the psyche. In particular, some of the healers interviewed pointed out that, in the case of infant helminthiasis, the gender of the healer being female, her tone of voice more submissive compared to a man and the prayer's more gentle cadence are very important contributors to the healing. This may be explained considering that the 'female figure which cures' is the embodiment in the child's subconscious of his mother or another close relative which provides beneficial effects for recovery from the illness. Often, saints and religious images are invoked but so too are natural ones (light, water, plants) [45]. Sometimes, it is the patient's 'belief' itself and his intense rapport with the healer which brings about the healing. In fact, both 'helpers' and community members affirm that only a 'believer' can be cured. Even though women seem to predominate in terms of their 'will power' to perform traditional healing, the ailments vary according to gender and age. For example, patients suffering from worms are usually young children brought to the healer by their mothers or grandmothers.

We would like to underline some parallels between therapeutic practises, the spells reported in this work and those described [18] in the area of Monte Vulture (Lucania) in the villages of Arbëreshë and Italians which would allow a comparison of their respective medical traditions. It should be clarified that Arbëreshë are the descendents of Southern Albanian immigrants who came to the south of Italy between the fifteenth and sixteenth centuries and who arrived in the Vulture area at the end of the 1600s [46].

As regards the helminthiasis rituals in Sicily it is reported that even in some Italian and Arbëreshë communities in Lucania the figure of 'healer' is seen as a wizard, witch or as 'helper'. In the area of Monte Vulture the type of healing is that which helps the patient overcome a period of illness during which the healer often resorts to 'magical' practises. At each step towards the cure, the therapy is personal and identifying the illness is prevalently based on physical signs [18].

In this area, many therapies which utilise rituals and folk medicine to cure the 'evil eye', erysipelas and infant intestinal parasites were examined. It would seem that helminthiasis in particular is diagnosed when the parasites are discovered in the faeces. These worms are thought to invade the whole body, moving around from intestines to heart to throat. To treat them, the healer uses scissors to make symbolic cuts on the abdomen in the form of crosses. At the same time, the following prayer is sung [18]:

"*Worm one, worm two, worm three, worm four, worm five, worm six, worm seven, worm eight, worm nine, Worm get out of this heart *(or Saint Saviour, get the worms and put them out).

This spell where 'papulo' means worm must be repeated three times. During each prayer nine symbolic cuts are made and at the end of the procedure the illness is often 'presented' to Saint Saviour. In Northern Italy, instead of making symbolic cuts with scissors, healers cut a piece of string which has been touched by the patient's body and which represents the worms within the body [47].

## Conclusion

Today in Sicily, as regards illness/health, it may be highlighted that over the last decade conventional medicine has been widely adopted with satisfying results as in recovery from cases of even mortal pathologies. In some social classes, 'conventional' and traditional medicine co-exist even though they do not really collaborate. It still holds true that the elder members of certain lower and less well-educated social groups consult 'healers' who practise rituals and use herbal concoctions which are both 'magical' and empirical to cure some pathologies.

Nowadays, interest in ancestral folk medicine in a civilised country like Italy only makes sense if the end is to recoup a cultural heritage that may be in irreversible decline. In reality, the reinforcement of industrial culture and the weakening of folk culture is increasingly the case, although it is still possible to find people in many areas who resort to traditional medical therapies. Such practices, fruit of the obsolete logic of an agricultural and pastoral society, might today seem primitive but they deserve respect and should be examined and re-evaluated from a medico-anthropological and socio-cultural point of view.

During the course of this research, magical beliefs and rituals particularly concerning helminthiasis have been documented and they have highlighted that although its aetiology – diagnosis and treatment – may not be accepted by 'official medicine', ancestral cures which amount to a psychotherapeutic effect could contribute to current medical therapy.

In the past, 'magical' illnesses and traditional cures were far more generally believed in than today. Nowadays, there is a raft of factors involved in the decline of traditional therapies among them, lessening religious fervour has reduced the psychotherapeutic efficacy of 'spells', 'signs' and rituals. Furthermore, the changing economic climate has brought about among younger and more educated men and women a disinterest in serving an apprenticeship as 'healers'.

Hospitals and clinics too, evermore highly equipped, with ever greater availability of specialised drugs, have lead the vanguard in the belief that empirical medicine was no longer necessary to eradicate many diseases among which the various intestinal parasites. Futhermore, the many psychotherapeutic treatments on offer have helped resolve a good number of 'magical' diseases or in some way those determined by psychic factors. Notwithstanding, it is important to note that magico-medical rituals persist from an anthropological-medical point-of-view above all considering this discipline as pivotal to a socio-anthropological interpretation of the medical act as a total doctor-patient relationship. Every clinical deed depends on twofold subjectivity – doctor-patient. When this relationship goes through a crisis the patient distances himself from the doctor in search of alternative solutions of the most diverse origin (ideological, psychological, socio-cultural, religious, magical-talismanic). When a person's equilibrium alters due to illness he can ask for help more or less explicitly to get over this event which is changing his existential models. At this point, a doctor must be willing to answer the patient's demands to overcome his temporary or chronic state of corporeal, human and social debilitation. Here, there is a fundamentally confidential role in the doctor-patient/diagnostic-therapeutic relationship which reduces the professional distance which all patients are insurmountably aware of. In the past, the doctor-patient approach was typically reciprocally discrete. Today, however, conditions are ripe for medical dialogue with the aim of optimising diagnostic and therapeutic effects.

From a medico-anthropological point of view, the persistence of the practises and rituals cited in this work may be due to the fact that medicine often communicates with its public without decoding its technical-scientific language making it often incomprehensible. The lack of linguistic and conceptual simplification, even more than that of professional shortcomings, can generate a lack of faith and a tendency to find 'refuge', remedies and security in practises and customs from the past. To complicate things, the need for a therapeutic rapport with a growing number of ethnic groups, will make the challenge of the 21^st ^century not only a scientific one but also an anthropological one.

## Appendix

Alphabetic list of the sicilian dialect denominations of named species and their corresponding popular and scientific names: in brackets, the botanical family and eventual synonyms.

*Addauru*, bay: *Laurus nobilis *L. (Lauraceae); *agghiu*, garlic: *Allium sativum *L. (Alliaceae); *aina picciridda*, corn cockle:*Agrostemma githago *L. (Caryophyllaceae); *alivu*, olive tree: *Olea europaea *L. var. *europaea *(Oleaceae); aruta, rue: Ruta chalepensis L. (Rutaceae); assìnziu-serìfiu, sea wormwood: Artemisia cretacea (Fiori) Pign. [(= A. maritima Bertol. (Asteraceae)]; *canfuridda*, wild chamomile: *Achillea ligustica *All. (Asteraceae); *cardedda *or *cardedda d'invernu*: snow thistle: *Sonchus oleraceus *L. (Asteraceae); *ceùsu jancu*, mulberry: *Morus alba *L. (Moraceae); *cipudda*, onion: *Allium cepa *L. (Alliaceae); *curaddina*, *simenza-ppi-li-viermi *or *simenza rê viermi*, coral moss: *Corallina officinalis *L. (Corallinaceae, Rhodophyta); *erva di vermi *or *erva atanasia*, tansy: *Tanacetum vulgare *L. (Asteraceae); *erva-ianca*, wormwood tree: *Artemisia arborescens *(Vaill.) L. (Asteraceae); *erva pucciddana*, green purslane, purslane *Portulaca oleracea *L. (Portulacaceae); *erva-sciàtica*, small flowered willow-weed: *Epilobium parviflorum *Schreb. (Onagraceae); *filicia *atirio, lady fern: *Athyrium filix-foemina *(L.) Roth (Athyriaceae); *fìlici masculina*, male-fern: *Dryopteris filix-mas *(L.) Scott (Aspidiaceae); *frastunaca*, wild carrot: *Daucus carota *L. (Apiaceae); *fraulara*, damascens nigella, love in a mist: *Nigella damascena *L. (Ranunculaceae); *majurana*, sweet marjoram: *Origanum majorana *(Lamiaceae); *marrùbbiu*, horehound: *Marrubium vulgare *L. (Lamiaceae); *menta*, menta, mint: *Mentha *× *piperita *L. (Lamiaceae); *nipitedda *o *nièbbita*: calamint [*Calamintha nepeta *(L.) Savi (Lamiaceae)]; *panicaudu*, snakeroot eryngo: *Eryngium campestre *L. (Apiaceae); *piricò*, St. John's wort: *Hypericum perforatum *L. (Clusiaceae); *puddisinu*, parsley: *Petroselinum sativum *Hoffm. = *P. crispum *(Mill.) A.W. Hill (Apiaceae); *rrosamarinu*, rosemary: *Rosmarinus officinalis *L. (Lamiaceae); *rrosa sarvaggia*, dog rose: *Rosa canina *L. (Rosaceae); *simienza di viermi *or *cabbarrasi*, stavesacre: *Delphinium staphysagria *L. (Ranunculaceae); *sudda sarvaggia*, *erva-ri-pittrògghiu*, scurfy pea: *Bituminaria bituminosa *(L.) E. H. Stirton (Fabaceae); *summaccu arbòriu*, tree of Heaven: *Ailanthus altissima *(Mill.) Swingle (Simaroubaceae); *tamareddu*, spanish origanum: [*Coridothymus capitatus *(L.) Rchb. (Lamiaceae)]; *tè sicilianu*, american wormseed: *Chenopodium ambrosioides *L. (Chenopodiaceae); *viti*, vine: *Vitis vinifera *L. subsp. *vinifera *(Vitaceae).

## Competing interests

The authors declare that they have no competing interests.
